# Longitudinal Changes in Isometric Trunk and Pelvic Strength Balance, Functional Movement and Muscle Imbalance During a Physiotherapy-Based Strengthening Programme in Adolescent Male Soccer Players

**DOI:** 10.3390/healthcare14111539

**Published:** 2026-06-01

**Authors:** Laura Zaliene, Jurgita Boltutiene, Rita Gikariene

**Affiliations:** 1 Department of Holistic Medicine and Rehabilitation, Faculty of Health Science, Klaipėda University, LT-92294 Klaipėda, Lithuania; 2 Department of Rehabilitation and Aesthetic therapy, Faculty of Health Science, Higher Education Institution, LT-91274 Klaipėda, Lithuania

**Keywords:** youth athletes, neuromuscular control, movement screening, isometric strength, postural stability, hip strength balance, sports physiotherapy, injury-risk monitoring

## Abstract

**Background:** Trunk and pelvic strength balance and functional movement quality are relevant factors for musculoskeletal health and injury-prevention monitoring in youth soccer players. **Aim:** This study aimed to evaluate longitudinal changes in isometric trunk and pelvic strength balance, functional movement quality, and selected muscle imbalance indicators during a physiotherapy-based strengthening programme in adolescent male soccer players. **Methods:** A longitudinal single-group repeated-measures study was conducted in male soccer players aged 12–18 years. Isometric strength balance was assessed using the Dr. Wolff Back-Check system, and functional movement quality was evaluated using the Functional Movement Screen (FMS). Complete-case analyses were performed according to available repeated measurements. **Results:** FMS total scores improved across repeated assessments, whereas the global Back-Check score showed no significant longitudinal change. Component-level and imbalance analyses indicated reductions in adductor–abductor imbalance, and better FMS performance was moderately associated with lower adductor–abductor imbalance. **Conclusions:** Functional movement quality and selected muscle imbalance indicators demonstrated favorable longitudinal changes during the physiotherapy-based strengthening programme. These findings suggest that physiotherapy-oriented strengthening and movement-control exercises may contribute to improvements in functional movement quality and selected muscle balance indicators in adolescent male soccer players. However, the small repeated-measures subsamples and observational study design limit causal interpretation and generalizability.

## 1. Introduction

Soccer is a high-intensity team sport characterized by sprinting, deceleration, changes in direction, jumping, kicking, and repeated player-to-player contact. These movement demands place substantial stress on the musculoskeletal system and are associated with a high burden of lower-extremity and trunk-related injuries in youth and adult players [1,2,3].

Trunk and pelvic musculature contribute to postural control, force transfer, and movement efficiency during soccer-specific tasks. Insufficient trunk strength altered neuromuscular control, and muscle imbalances may impair movement quality and have been linked to low back pain, lower-extremity overload, and reduced athletic readiness [4,5,6,7,8]. In adolescent athletes, these factors are especially relevant because rapid growth, training specialization, and incomplete neuromuscular maturation may amplify functional asymmetries and instability.

Sport-specific assessment is therefore important not only for performance monitoring but also for identifying deficits that may be amenable to targeted intervention. The Dr. Wolff Back-Check system enables standardized isometric assessment of trunk and lower-limb muscle groups and provides clinically useful information on strength balance and imbalance patterns [9,10,11]. In parallel, the Functional Movement Screen (FMS) is widely used to characterize movement quality and detect deficits in mobility, stability, and motor control [12,13,14,15]. From a physiotherapy perspective, combining movement-quality screening with objective strength-balance assessment may provide a more comprehensive profile of functional readiness than either measure alone. While FMS reflects integrated movement patterns, mobility, and motor control, the Back-Check assessment provides more specific information on isometric trunk and pelvic muscle balance. This combined approach may be particularly relevant in adolescent soccer players, in whom growth-related neuromuscular variability and sport-specific loading may contribute to asymmetries that are not fully captured by isolated screening tools.

Although core strengthening and neuromuscular training programs may improve movement quality and selected performance outcomes in soccer players [16,17,18,19,20,21], objective monitoring of trunk and pelvic strength balance is still not routinely integrated into youth soccer prevention practice. Moreover, the relationship between functional movement quality and specific muscle imbalance indices remains insufficiently described in adolescent soccer players.

Therefore, the aim of this study was to investigate longitudinal changes in isometric trunk and pelvic strength balance, functional movement quality, and selected muscle imbalance indicators during a physiotherapy-based strengthening programme in adolescent male soccer players, as well as associations between functional movement quality and Back-Check-derived imbalance measures.

## 2. Materials and Methods

### 2.1. Study Design and Participants

This study used a longitudinal single-group repeated-measures design to evaluate changes in functional movement quality, isometric trunk and pelvic strength balance, and muscle imbalance variables during a physiotherapy-based strengthening programme in adolescent male soccer players from a soccer school/academy setting. Assessments were conducted in both the Neurosensomotorics Laboratory and the training environment. For the present manuscript, complete-case analyses were performed separately for each outcome measure, as not all participants completed all repeated assessments. Consequently, analytic subsamples were defined as participant groups based on the number of completed repeated assessments available for each measurement instrument. The assessment schedule included three potential measurement time points: baseline assessment (T0), first follow-up assessment (T1), and second follow-up assessment (T2), conducted during the physiotherapy-based training period. For the Back-Check, the subsamples comprised *n* = 25 at baseline only (T0), *n* = 21 with T0 and T1 data, and *n* = 13 with T0, T1, and T2 data. For FMS, the corresponding subsamples comprised *n* = 27 at baseline only, *n* = 23 with T0 and T1 data, and *n* = 7 with T0, T1, and T2 data. Inclusion criteria were male sex, age 12–18 years, active participation in organized soccer training, medical clearance for full participation in training and testing, and the ability to complete all required assessment procedures. Exclusion criteria were acute musculoskeletal injury, pain or medical condition limiting maximal effort or functional movement testing on the assessment day, current rehabilitation after injury that restricted full training participation, and incomplete raw data for the relevant analysis. Written informed consent was obtained from parents/legal guardians, and assent was obtained from all participants. The study was conducted in accordance with the Declaration of Helsinki and approved by the Klaipėda State University of Applied Sciences Ethics Committee (approval No. SSV6-114; approval date: 27 December 2024).

### 2.2. Back-Check Assessment

Isometric muscle strength balance was assessed using the Back-Check Sports & Prevention device (Dr. Wolff, Arnsberg, Germany). The Back-Check system has previously been used for standardized assessment of trunk and muscle strength balance in athletic populations [9,10,11]. Assessments were performed according to the manufacturer’s standardized testing procedures. In accordance with the manufacturer’s guidelines, participants were positioned in an upright stance with slightly flexed knees while the pelvis was stabilized using adjustable support pads. The best of three maximal efforts was recorded for each movement. The assessment protocol comprised the overall Back-Check Score (BCS) as well as the following component measures: cervical strength (CS), upper body strength (UBS), lower body strength (LBS), combined lateral flexion (CLF), adductor strength (ADD), abductor strength (ABD), core extension/flexion, press/pull, and hip extension/flexion. Muscle strength values obtained from the Back-Check device were recorded according to the manufacturer’s standardized output system and expressed in kilograms of force (kg). For analytical purposes, both individual component values and composite balance indicators were evaluated.

### 2.3. Functional Movement Assessment

Functional movement quality was assessed using the Functional Movement Screen. The Functional Movement Screen (FMS) is a standardized movement assessment tool commonly used to evaluate mobility, stability, and movement asymmetries in athletic populations [12,13,14,15]. All FMS assessments were performed according to standardized scoring procedures described in the previous literature. The seven standard subtests (deep squat, hurdle step, lunge, shoulder mobility, active straight-leg raise, push-up, and rotary stability) were scored on the conventional 0-3 scale, and a total score was calculated as the sum of the subtests. In line with previous literature and prior practice in the project, an FMS total score ≤ 14 was used descriptively as an elevated injury-risk indicator and was not used as a primary statistical outcome measure.

### 2.4. Derived Imbalance Variables

To evaluate segment-specific muscle imbalance, absolute difference scores were calculated for selected pairs of variables: adductor-abductor imbalance (ADD–ABD), core imbalance (core extension/flexion difference), and press-pull imbalance (press/pull difference). Imbalance variables were calculated as absolute differences between paired muscle strength values obtained from the Back-Check assessment (expressed in kg), with higher values indicating greater asymmetry between opposing muscle groups. These derived variables were selected because they reflect clinically relevant asymmetries that may not be captured by the global Back-Check score alone.

### 2.5. Intervention Context

During the study period, players participated in a physiotherapy-based strengthening programme conducted in the soccer training environment under physiotherapy supervision. The programme was integrated into the athletes’ regular training routine and implemented throughout the assessment period. Participants included in the repeated-measures analyses participated regularly in the physiotherapy-based strengthening sessions conducted throughout the intervention period; however, detailed attendance records were not systematically available for all participants. Exercise sessions were performed 2–3 times per week, with each session lasting approximately 20–30 min. Exercise intensity and progression were adjusted individually according to movement quality, technical execution, and participant tolerance, using body-weight and functional strengthening exercises. Sessions focused on trunk stabilization, pelvic control, hip adductor and abductor strengthening, abdominal and back extensor activation, and movement-quality exercises aimed at improving neuromuscular control and functional movement patterns. The programme included corrective, stabilization, and functional strengthening exercises using body-weight and functional training approaches. Because attendance and repeated testing completeness varied across players, the present analyses emphasize within-subsample longitudinal changes rather than direct comparisons between formally balanced intervention groups.

### 2.6. Statistical Analysis

Descriptive statistics are presented as mean ± standard deviation, together with median and range where relevant. The normality of continuous variables was assessed using visual inspection and normality testing. When distributional assumptions were not met or sample sizes were small, non-parametric tests were prioritized for inferential interpretation. Due to incomplete follow-up and variability in repeated assessments, complete-case analyses were performed separately for each outcome measure. An intention-to-treat approach was not applied because the study did not include randomized allocation or controlled intervention groups. Statistical comparisons were based on continuous FMS total scores, whereas the proportion of players with FMS ≤ 14 was reported descriptively as an elevated injury-risk indicator. A formal a priori sample size calculation was not performed because the study was based on an available cohort of academy players participating in the physiotherapy-based monitoring programme. Paired-samples *t*-tests were reported only as supplementary analyses where previously calculated and were not used as the primary basis for interpretation when non-parametric assumptions were more appropriate. For three repeated measurements, Friedman tests were used, with Kendall’s W reported as an effect size. Spearman correlation coefficients were calculated to assess associations between FMS and Back-Check-derived imbalance variables. Statistical significance was set at *p* < 0.05. Given the small repeated-measures subsamples, non-parametric analyses were considered the primary inferential approach. Additional baseline comparisons between completers and non-completers were performed to evaluate potential attrition-related selection bias.

## 3. Results

### 3.1. Descriptive Characteristics of Participants

The analytic sample structure is presented in Table 1. The analytic sample structure differed by instrument because of incomplete follow-up. For Back-Check analyses, the baseline-only subsample included 25 players, the two-time-point subsample included 21 players, and the three-time-point subsample included 13 players. For FMS analyses, the corresponding subsamples included 27, 23, and 7 players, respectively.

### 3.2. Functional Movement Screen Outcomes

FMS outcomes are summarized in Table 2. In the baseline-only FMS subsample (*n* = 27), the mean FMS total score at T0 was 17.74 ± 2.65 points. In the two-time-point subsample (*n* = 23), the mean FMS score increased from 16.57 ± 1.70 at T0 to 18.52 ± 2.04 at T1. This change was statistically significant according to the Wilcoxon signed-rank test (*p* = 0.0008), and the paired-samples *t*-test also indicated a significant increase (t(22) = 4.42, *p* = 0.00021; Cohen’s d = 0.92).

In the three-time-point FMS subsample (*n* = 7), the mean FMS score increased from 16.43 ± 1.51 at T0 to 17.14 ± 1.07 at T1 and 19.14 ± 1.46 at T2. The Friedman test showed a statistically significant difference across time points (chi-square = 11.19, *p* = 0.0037; Kendall’s W = 0.80). Pairwise comparisons showed no significant difference between T0 and T1 (*p* = 0.531), whereas significant improvements were observed between T0 and T2 (*p* = 0.0156) and between T1 and T2 (*p* = 0.0156).

Descriptively, the prevalence of elevated injury risk defined by FMS ≤ 14 was 11.1% in the baseline-only subsample, 8.7% at both T0 and T1 in the two-time-point subsample, and declined from 14.3% at T0 to 0% at T1 and T2 in the three-time-point subsample.

### 3.3. Back-Check Global Score and Component Outcomes

Back-Check outcomes are presented in Table 3A,B. In the baseline-only Back-Check subsample (*n* = 25), the mean BCS at T0 was 3.24 ± 1.44 points (median 3.10; range 1.2–6.9). In the two-time-point subsample (*n* = 21), BCS increased from 2.98 ± 1.58 at T0 to 3.25 ± 1.29 at T1, but this change was not statistically significant (Wilcoxon *p* = 0.539; paired *t*-test *p* = 0.491; Cohen’s *d* = 0.15).

In the three-time-point Back-Check subsample (*n* = 13), BCS values were 3.79 ± 0.96 at T0, 3.68 ± 0.92 at T1, and 4.03 ± 1.17 at T2. The Friedman test showed no statistically significant difference across the three time points (chi-square = 2.63, *p* = 0.269; Kendall’s W = 0.10). Pairwise comparisons were also non-significant (T0 vs. T1 *p* = 0.424; T0 vs. T2 *p* = 0.530; T1 vs. T2 *p* = 0.292).

At the component level, the only statistically significant change in the two-time-point subsample was observed for CLF, which increased from T0 to T1 (*p* = 0.035; mean change +2.44; dz = 0.55). In the three-time-point subsample, no single component demonstrated a statistically significant overall Friedman test across all three time points. However, pairwise analyses indicated a significant increase in ADD between T0 and T2 (*p* = 0.0195), whereas comparisons between T0 and T1 for ADD (*p* = 0.0547) and CS (*p* = 0.0574) did not reach statistical significance.

### 3.4. Muscle Imbalance Analysis

Muscle imbalance outcomes are shown in Table 4A,B. Adductor-abductor imbalance decreased significantly in both repeated-measures Back-Check subsamples. In the two-time-point subsample, ADD–ABD imbalance declined from 3.42 ± 2.91 at T0 to 2.11 ± 2.34 at T1 (*p* = 0.041). In the three-time-point subsample, it declined progressively from 4.87 ± 3.12 at T0 to 3.95 ± 2.88 at T1 and 2.76 ± 2.41 at T2 (*p* = 0.038; Kendall’s W = 0.31).

Core imbalance and press-pull imbalance also decreased over time, but these changes did not reach statistical significance. Core imbalance declined from 3.98 ± 3.44 to 2.85 ± 2.96 in the two-time-point subsample (*p* = 0.089) and from 4.26 ± 3.02 to 3.11 ± 2.55 to 2.94 ± 2.18 in the three-time-point subsample (*p* = 0.072). Press-pull imbalance decreased from 3.76 ± 3.21 to 2.9 ± 2.67 in the two-time-point subsample (*p* = 0.118) and from 4.91 ± 3.44 to 3.88 ± 3.01 to 3.21 ± 2.73 in the three-time-point subsample (*p* = 0.095).

### 3.5. Correlation Between Functional Movement and Muscle Imbalance

Correlations are presented in Table 5. Spearman correlation analysis demonstrated a statistically significant inverse association between FMS total score and adductor-abductor imbalance at baseline (rho = −0.46, *p* = 0.018), indicating that better functional movement quality was associated with lower muscle imbalance. A stronger inverse relationship was observed between FMS change and change in adductor-abductor imbalance in the repeated-measures subsample (rho = −0.52, *p* = 0.021). Correlations between FMS and the remaining imbalance indicators were weak and not statistically significant.

### 3.6. Completer and Non-Completer Baseline Comparison

Baseline comparisons were performed to evaluate potential selection bias associated with incomplete follow-up. For the FMS analyses, participants who completed all three assessments (*n* = 7) demonstrated similar baseline FMS total scores compared with non-completers (16.43 ± 1.51 vs. 16.60 ± 1.76, respectively; Mann–Whitney U test, *p* = 0.735). However, completers were older on average than non-completers (16.71 ± 0.95 vs. 14.80 ± 1.94 years, respectively; *p* = 0.027).

Similarly, in the Back-Check analyses, participants who completed all three assessments (*n* = 13) demonstrated somewhat higher baseline BCS values compared with non-completers (3.79 ± 0.96 vs. 2.98 ± 1.58, respectively), although this difference was not statistically significant (*p* = 0.099). Completers were also older on average than non-completers (16.62 ± 0.77 vs. 14.14 ± 1.77 years, respectively; *p* < 0.001). These findings suggest that although baseline functional and strength-balance measures were not significantly different between groups, age-related developmental differences and incomplete follow-up may still have influenced the longitudinal findings and reduced generalizability.

## 4. Discussion

The principal finding of this study was that functional movement quality improved across repeated assessments, whereas global isometric strength-balance scores remained relatively stable over time. This apparent divergence became more informative when segment-specific imbalance variables were examined. In particular, adductor–abductor imbalance decreased significantly, and this reduction showed a moderate association both with higher Functional Movement Screen (FMS) scores and with improvements in FMS performance over time.

The observed changes in FMS scores suggest that movement quality in adolescent soccer players may improve during a physiotherapy-oriented training period, especially when players are monitored longitudinally across multiple assessments. This interpretation is consistent with previous findings indicating that targeted functional and neuromuscular training programs can enhance FMS performance and address specific movement deficits in soccer players and other athletic populations [12,13,14,15,16,18,19,20,21]. In the present dataset, the largest FMS-related effect was observed in the subsample assessed at three time points, in which the prevalence of elevated injury risk—based on the conventional FMS cut-off—decreased to zero by the final follow-up. However, given the small sample size and the descriptive rather than prospective nature of injury monitoring, this finding should be interpreted with caution.

The outcome-specific nature of exercise-based adaptations should also be considered. Evidence from other youth sport populations suggests that complementary training approaches do not always result in measurable improvements across all physical fitness outcomes. For example, Greco et al. reported no significant differences in flexibility or vertical jump performance between young female volleyball players practicing Pilates and those following standard volleyball training [22]. Although the sport, sex, and intervention differ from the present study, these findings support the need for outcome-specific monitoring and cautious interpretation of physiotherapy- or exercise-based adaptations in adolescent athletes.

In contrast to the improvements observed in functional movement quality, the global Back-Check score did not demonstrate statistically significant change over time. Because global summary scores may not fully capture segment-specific adaptations, imbalance analyses were additionally performed using absolute difference scores. The observed increase in combined lateral flexion strength and the progressive reduction in adductor–abductor imbalance indicate that localized neuromuscular adaptations can occur even when changes in overall composite scores are modest. The greater responsiveness of adductor–abductor imbalance may be related to the programme’s emphasis on pelvic control, hip stabilization, and targeted activation of the hip adductor and abductor muscle groups. These exercises may have more directly influenced frontal-plane hip control than global trunk or press–pull strength components. From a clinical standpoint, these findings support the interpretation of Back-Check results at the component and imbalance level, rather than reliance on a single summary metric.

One potentially relevant finding of the present study is the inverse association between FMS performance and adductor–abductor imbalance. This relationship suggests that higher-quality movement patterns are not merely reflected in composite screening scores but may also correspond to more favorable segmental muscle balance, particularly in the pelvic and hip regions. Given the functional demands placed on the hip adductors in soccer and the high prevalence of groin-related symptoms reported in this population, this association may have practical relevance for screening and targeted exercise prescription [2,3,7,17].

Several limitations of the study should be acknowledged. First, different complete-case subsamples were available for the FMS and Back-Check analyses because of incomplete follow-up across repeated assessments, which limited direct one-to-one comparisons across all outcomes. This complete-case approach may have introduced selection bias and reduced the internal validity of longitudinal comparisons. Second, the smallest repeated-measures FMS subsample comprised only seven participants, reducing statistical power and the precision of effect estimates. Third, exposure to the physiotherapy-oriented training intervention was not fully standardized across players; accordingly, the present analysis emphasizes within-subsample longitudinal patterns rather than definitive causal inferences between groups. Detailed intervention adherence data were not available for all participants, limiting the ability to quantify individual training exposure across the study period. Fourth, some Back-Check component variables contained missing values or software-generated non-calculable outputs, which were treated as missing in the analyses. Additional baseline comparisons between completers and non-completers demonstrated generally similar baseline functional movement and Back-Check outcomes, although completers were older on average. Consequently, developmental and maturational differences may have influenced the observed longitudinal patterns despite the absence of major baseline performance disparities. Finally, although injury characteristics were recorded descriptively, the study was not designed or powered as a prospective injury-prediction investigation. In addition, the observational longitudinal design limits causal interpretation regarding the effects of the physiotherapy-based strengthening programme.

Despite these limitations, the findings provide clinically relevant insights for physiotherapy practice and youth soccer development. The combined use of FMS and Back-Check–derived imbalance metrics may facilitate the identification of functional deficits that are not apparent from global scores alone. In particular, monitoring adductor–abductor balance, core control, and overall movement quality may be valuable for informing individualized preventive and corrective exercise programs in adolescent soccer players.

## 5. Conclusions

Functional movement quality improved significantly across repeated assessments in the analyzed complete-case subsamples, whereas no statistically significant longitudinal change was observed in the global Back-Check score during the physiotherapy-based strengthening programme. However, component-level analyses demonstrated reductions in adductor–abductor muscle imbalance and localized functional adaptations not fully reflected by the global composite score. In addition, better functional movement quality was moderately associated with lower adductor–abductor imbalance. These findings suggest that physiotherapy-oriented strengthening and movement-control exercises may contribute to improvements in functional movement quality and selected muscle balance indicators in adolescent male soccer players. Nevertheless, the small repeated-measures subsamples and observational study design limit causal interpretation and generalizability.

## Figures and Tables

**Table 1:** Analytic sample structure and age characteristics.

Outcome	Subsample	*n*	Age (Years, Mean ± SD)
Back-Check	Baseline only	25	14.8 ± 1.9
Back-Check	Two time points	21	14.7 ± 1.8
Back-Check	Three time points	13	16.9 ± 1.1
FMS	Baseline only	27	14.6 ± 1.9
FMS	Two time points	23	14.6 ± 1.9
FMS	Three time points	7	16.7 ± 1.0

**Table 2:** Functional Movement Screen outcomes across repeated assessments.

Subsample	Time	*n*	FMS Total (Mean ± SD)	Median (IQR)	Min–Max	FMS ≤ 14 *n* (%)	Statistical Comparison
Baseline only	T0	27	17.74 ± 2.65	18 (16–20)	11–21	3 (11.1)	-
Two time	T0	23	16.57 ± 1.70	17 (15–18)	12–19	2 (8.7)	Wilcoxon signed-rank test: *p* = 0.0008, r = 0.65
	T1	23	18.52 ± 2.04	19 (17–20)	14–20	2 (8.7)	
Three time points	T0	7	16.43 ± 1.51	17 (15–17)	14–18	1 (14.3)	Friedman test: *p* = 0.0037, Kendall’s W = 0.80
	T1	7	17.14 ± 1.07	17 (16–18)	15–18	0 (0)	Post hoc comparisons: T0 vs. T1 *p* = 0.531, r = 0.12; T0 vs. T2 *p* = 0.0156, r = 0.71; T1 vs. T2 *p* = 0.0156, r = 0.69
	T2	7	19.14 ± 1.46	20 (18–20)	16–20	0 (0)	

Abbreviations: FMS, Functional Movement Screen; IQR, interquartile range; SD, standard deviation; r, effect size for Wilcoxon pairwise comparisons.

**Table 3:** Back-Check outcomes.

**(A) Back-Check Outcomes in the Two-Time-Point Subsample**						
**Variable**	**T0 (Mean ± SD)**	**T1 (Mean ± SD)**	**Wilcoxon** ***p***		**Effect Size**	
BCS	2.98 ± 1.58	3.25 ± 1.29	0.539		0.15	
CLF	1.72 ± 2.61	4.16 ± 4.03	0.035		0.55	
**(B) Back-Check Outcomes in the Three-Time-Point Subsample**						
**Variable**	**T0 (Mean ± SD)**	**T1 (Mean ± SD)**	**T2 (Mean ± SD)**	**Friedman** ***p***	**Kendall’s W**	**Post Hoc Comparisons**
BCS	3.79 ± 0.96	3.68 ± 0.92	4.03 ± 1.17	0.269	0.1	T0 vs. T1: *p* = 0.424, r = 0.22; T0 vs. T2: *p* = 0.530, r = 0.16; T1 vs. T2: *p* = 0.292, r = 0.28
CS	4.38 ± 1.46	3.38 ± 0.75	4.27 ± 1.80	0.063	0.39	T0 vs. T1: *p* = 0.057, r = 0.52
ADD	0.76 ± 1.94	3.32 ± 3.33	5.35 ± 3.64	0.15	0.27	T0 vs. T2 *p* = 0.0195, r = 0.67

Abbreviations: BCS, Back-Check Score; CS, cervical/core strength; CLF, combined lateral flexion; ADD, adductor strength; SD, standard deviation; r, effect size for pairwise comparisons.

**Table 4:** Muscle imbalance outcomes.

**(A) Muscle Imbalance Outcomes in the Two-Time-Point Subsample**						
**Imbalance Variable**	**T0 (Mean ± SD)**	**T1 (Mean ± SD)**	**Wilcoxon** ***p***		**Effect Size**	
ADD–ABD imbalance	3.42 ± 2.91	2.11 ± 2.34	0.041		0.45	
Core imbalance	3.98 ± 3.44	2.85 ± 2.96	0.089		0.37	
Press-pull imbalance	3.76 ± 3.21	2.94 ± 2.67	0.118		0.31	
**(B) Muscle Imbalance Outcomes in the Three-Time-Point Subsample**						
**Imbalance Variable**	**T0 (Mean ± SD)**	**T1 (Mean ± SD)**	**T2 (Mean ± SD)**	**Friedman *p***	**Kendall’s W**	**Post Hoc Comparisons**
ADD–ABD imbalance	4.87 ± 3.12	3.95 ± 2.88	2.76 ± 2.41	0.038	0.31	T0 vs. T1: *p* = 0.219, r = 0.34; T0 vs. T2: *p* = 0.031, r = 0.61; T1 vs. T2: *p* = 0.125, r = 0.41
Core imbalance	4.26 ± 3.02	3.11 ± 2.55	2.94 ± 2.18	0.072	0.24	T0 vs. T1: *p* = 0.156, r = 0.39; T0 vs. T2: *p* = 0.094, r = 0.47
Press-pull imbalance	4.91 ± 3.44	3.88 ± 3.01	3.21 ± 2.73	0.095	0.21	T0 vs. T1: *p* = 0.188, r = 0.36; T0 vs. T2: *p* = 0.109, r = 0.44

Abbreviations: ADD–ABD, adductor–abductor; SD, standard deviation; r, effect size for pairwise comparisons.

**Table 5:** Spearman correlations between FMS and muscle imbalance indicators.

Variable Pair	rho	*p*	Interpretation
FMS total vs. ADD–ABD imbalance	−0.46	0.018	Moderate inverse association
FMS total vs. core imbalance	−0.31	0.112	Weak, non-significant
FMS total vs. press-pull imbalance	−0.27	0.174	Weak, non-significant
FMS change vs. ADD–ABD change	−0.52	0.021	Moderate-to-strong inverse association
FMS change vs. core imbalance change	−0.34	0.141	Weak, non-significant
FMS change vs. press-pull imbalance change	−0.29	0.201	Weak, non-significant

Abbreviations: FMS, Functional Movement Screen; rho, Spearman correlation coefficient.

## Data Availability

The data presented in this study are available from the corresponding author upon reasonable request. The data are not publicly available due to privacy restrictions.
